# The integrity of the U12 snRNA 3′ stem–loop is necessary for its overall stability

**DOI:** 10.1093/nar/gkab048

**Published:** 2021-02-12

**Authors:** Antto J Norppa, Mikko J Frilander

**Affiliations:** Institute of Biotechnology, P.O. Box 56, Viikinkaari 5, University of Helsinki, FI-00014 Helsinki, Finland; Institute of Biotechnology, P.O. Box 56, Viikinkaari 5, University of Helsinki, FI-00014 Helsinki, Finland

## Abstract

Disruption of minor spliceosome functions underlies several genetic diseases with mutations in the minor spliceosome-specific small nuclear RNAs (snRNAs) and proteins. Here, we define the molecular outcome of the U12 snRNA mutation (84C>U) resulting in an early-onset form of cerebellar ataxia. To understand the molecular consequences of the U12 snRNA mutation, we created cell lines harboring the 84C>T mutation in the U12 snRNA gene (*RNU12*). We show that the 84C>U mutation leads to accelerated decay of the snRNA, resulting in significantly reduced steady-state U12 snRNA levels. Additionally, the mutation leads to accumulation of 3′-truncated forms of U12 snRNA, which have undergone the cytoplasmic steps of snRNP biogenesis. Our data suggests that the 84C>U-mutant snRNA is targeted for decay following reimport into the nucleus, and that the U12 snRNA fragments are decay intermediates that result from the stalling of a 3′-to-5′ exonuclease. Finally, we show that several other single-nucleotide variants in the 3′ stem-loop of U12 snRNA that are segregating in the human population are also highly destabilizing. This suggests that the 3′ stem-loop is important for the overall stability of the U12 snRNA and that additional disease-causing mutations are likely to exist in this region.

## INTRODUCTION

Defects in pre-mRNA splicing are increasingly recognized as important contributors to human disease. While the majority of disease-causing mRNA splicing defects result from mutations in *cis*-acting signals necessary for intron recognition, a large number of disease-causing mutations have also been identified in the core components of the spliceosome and auxiliary splicing factors. A significant portion of human disease-causing mutations in the core spliceosome components have been described in the minor spliceosome. The minor spliceosome, also called the U12-dependent spliceosome, is a pre-mRNA splicing machinery that recognizes and excises <0.5% of introns in the human genome, while the major spliceosome (U2-dependent spliceosome) excises >99% of the introns. Both spliceosomes coexist in the cells of most metazoan species and show similar overall architecture, assembly pathways, and catalytic mechanisms. The main architectural difference between the two spliceosomes lies in the composition of small nuclear ribonucleoproteins (snRNPs) and specifically in the composition of their small nuclear RNA (snRNA) components. U1, U2, U4 and U6 snRNAs/snRNPs are unique to the major spliceosome while the respective U11, U12, U4atac and U6atac snRNAs/snRNPs are specific to the minor spliceosome (1–5). In contrast, the U5 snRNP is shared between the two spliceosomes. Despite divergence at the sequence level, analogous snRNAs in the two spliceosomes fold into similar secondary structures (3).

Minor spliceosome-specific snRNAs are present as single-copy genes in mammalian genomes, unlike the corresponding genes encoding snRNA components of the major spliceosome that are present as multigene arrays/families (6). Additionally, mammalian genomes also code for variants of all spliceosomal snRNAs that divert from ‘canonical’ snRNA sequences, and in most cases are of unknown functional significance (7). In addition to four unique snRNAs, the minor spliceosome contains a set of proteins not present in the major spliceosome (8,9), and vice versa, the majority of spliceosomal proteins are thought to associate with both spliceosomes. Although the overall splicing chemistry is similar between the two spliceosomes, intron recognition in the major spliceosome is carried out by individual U1 and U2 snRNPs, while the minor spliceosome analogs U11 and U12 function together as an intron recognition complex known as the U11/U12 di-snRNP (10,11). Consistent with the different mechanisms of intron recognition, the protein components known to be unique to one of the spliceosomes are found in the U11/U12 di-snRNP and the U1 and U2 snRNPs (8,9).

Mutations in both protein and snRNA components that are specific to the minor spliceosome have been linked to five hereditary diseases while one disease is caused by somatic mutations (12). In the majority of inherited diseases, the patients are compound heterozygotes carrying two separate mutations in the same locus. However, only with two diseases the underlying mechanisms have been explored. One of those is a member of a group of three diseases, which are all caused by mutations in the *RNU4ATAC* locus, encoding U4atac snRNA. Depending on the mutations, the patients suffer from any of three distinct disorders with partially overlapping phenotypes: Microcephalic Osteodysplastic Primordial Dwarfism type I, also known as Taybi–Linder syndrome (MOPD I/TALS; 13,14), Roifman syndrome (RFMN; 15) and Lowry Wood syndrome (LWS; 16). In this case, Jafarifar *et al.* (17) carried out an extensive analysis of point mutations related to MOPD I/TALS, and reported that the majority of the mutations disrupt the binding site of the 15.5K protein, leading to disruption of the U4atac/U6atac.U5 tri-snRNP. Additionally, one of the mutations leads to reduced U4atac snRNA levels presumably due to disruption of the binding site for Sm proteins (Sm site). In the other case, we investigated compound heterozygous mutations in the *RNPC3* gene, encoding the U11/U12-65K protein, that result in isolated growth hormone deficiency (IGHD; 18,19). We found that the disease is an outcome of two effects: reduction of mRNA levels due to nonsense-mediated decay, and reduced affinity of the U11/U12-65K protein to U12 snRNA because of a protein folding defect (19). Together these lead to reduced U11/U12 di-snRNP levels, causing a splicing defect.

**Figure 1.**
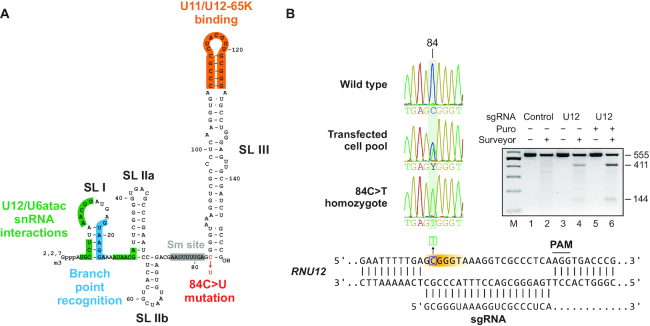
Construction of a HEK293 cell line model of the 84C>T U12 snRNA mutation. (**A**) Secondary structure and functional elements of human U12 snRNA. (**B**) Introducing the 84C>T mutation into the *RNU12* gene using base editing. *Bottom:* The single guide RNA (sgRNA) and the PAM site used for editing the *RNU12* gene are shown. The orange gradient marks the window where cytosine deamination is expected to take place using the Target-AID method (highest efficiency at -17 to -19 upstream of the PAM site). *Left:* Sequencing chromatograms of the deamination site from wild-type HEK293 cells, cells transfected with the Target-AID editing plasmid and selected with puromycin and a single-cell clone homozygous for the 84C>T mutation. *Right:* Analysis of the *RNU12* locus by Surveyor assay in HEK293 cell pools transfected with a modified Target-AID plasmid carrying negative control (Chinese hamster *hprt*; lanes 1–2) or *RNU12* sgRNA (lanes 3–4) and optionally selected with puromycin (lanes 5–6). Digestion of heteroduplexes with a mismatch at position +84 of the *RNU12* gene is expected to result in fragments of 411 bp and 144 bp.

Recently, an 84C>T mutation in the U12 snRNA gene (*RNU12*) has been shown to cause an early-onset form of cerebellar ataxia (EOCA) with autosomal recessive inheritance (20). EOCA patients that are homozygous for the 84C>T mutation display hypotonia at infancy, speech and learning difficulties, delayed motor development, ataxia, and cerebellar hypoplasia and degeneration (20). Interestingly, the 84C>U mutation is located a few nucleotides downstream of the Sm site at the base of the long 3′ terminal stem-loop III (SLIII) of U12 snRNA. Therefore, the mutation is located away from the key functional elements of U12 snRNA that interact with the branch point sequence of the intron and the U6atac snRNA during splicing (Figure 1A). The SLIII itself is important for the stability of the U11/U12 di-snRNP complex, as it is bound by the U11/U12-65K protein that serves as the bridge between U11 and U12 snRNPs (18,19,21,22). Consistently, the U11/U12 di-snRNP complex is disrupted by IGHD-causing mutations in the U11/U12-65K protein (18); however, location of the 84C>U U12 snRNA mutation does not suggest a similar molecular mechanism, as the binding site for the 65K protein is predicted to remain intact.

Here, we investigate the mechanistic consequences of the U12 84C>U mutation that causes early-onset cerebellar ataxia. We show that this mutation leads to accelerated decay of the U12 snRNA in several human cell lines. Furthermore, we detect several stable decay intermediates that lack the SLIII of U12 snRNA in cells carrying the disease mutation. Interestingly, the same decay intermediates can also be detected at low levels in wild-type cells, suggesting that the same decay process is part of the natural biogenesis or turnover of U12 snRNA. Additionally, we analyzed U12 variants carrying single nucleotide point mutations within the SLIII region. We identified several U12 SNP variants segregating in the human population that destabilize the U12 snRNA similarly to the EOCA mutation. This suggests that there may be additional U12 mutations leading to EOCA or similar human diseases.

## MATERIALS AND METHODS

### Plasmid construction

To create the pUC19-RNU12 plasmid, the *RNU12* gene, including 942 bp of upstream and 343 bp of downstream sequence was amplified from HEK293 genomic DNA using primers RNU12-BamHI-F and RNU12-HindIII-R and cloned into the BamHI and HindIII sites of the pUC19 vector. Subsequently, 15 extra nucleotides (5′-ATGAGATAGTGATAC-3′) were inserted into the 5′ end of the U12 snRNA sequence by PCR, resulting in pUC19-RNU12-5′tag. The 84C>T mutation and other mutations were introduced into the pUC19-RNU12-5′tag plasmid by site-directed mutagenesis. To create Target-AID-RNU12 plasmid, we used whole-plasmid PCR to replace the existing guide RNA sequence in plasmid pcDNA3.1_pCMV-nCas-PmCDA1-ugi pH1-gRNA(HPRT) with a guide RNA targeting the *RNU12* gene (GCGGGTAAAGGTCGCCCTCA). The original plasmid (Addgene #79620) was a gift from Dr. Akihiko Kondo. To enable more efficient enrichment of transfected cells, the G418 cassette in this plasmid was deleted by PCR, with simultaneous introduction of a *Bsr*GI site. Subsequently a puromycin cassette was cloned into the plasmid using *Xba*I and *Bsr*GI sites.

### Cell culture, treatments and transfection

HEK293 and HeLa cells were cultured in DMEM supplemented with 10% FBS, 1% penicillin–streptomycin and 2 mM L-glutamine. Plasmid transfections were carried out using Lipofectamine 2000 (Thermo Fisher). For tag-U12 experiments, each pUC19-RNU12-5′tag construct was cotransfected at a 3:1 ratio with pAVU6+27-F30-2xdBroccoli (Addgene #66842), a gift from Dr Samie Jaffrey. siRNA reverse transfections were carried out using Lipofectamine RNAiMAX (Thermo Fisher) and 30 nM final concentration of siRNA, followed by a 48 h incubation before RNA extraction with Trizol. siRNAs were purchased from Dharmacon or Sigma-Aldrich and their sequences are listed in Supplementary Table S1. For RNA half-life measurement, HEK293 cells at ∼50% confluency were treated with 5 μg/ml actinomycin D (Sigma-Aldrich) and lysed with Trizol at the indicated time points.

### Genome editing using the Target-AID method

HEK293 cells were transfected with 4 μg of the Targed-AID-puro-RNU12 plasmid using Lipofectamine 2000. Puromycin selection was applied at a 3 μg/ml final concentration for 72 h, starting 24 h after transfection. After selection, genomic DNA was extracted using the NucleoSpin Tissue kit (Macherey–Nagel). The *RNU12* locus was amplified using primers U12-surv-F and U12-surv-R (Supplementary Table S1). Editing was detected using the Surveyor Mutation Detection Kit (IDT) according to the manufacturer's instructions and confirmed by Sanger sequencing. Single-cell clones were obtained by limiting dilution cloning in 96-well plates, expanded to 12-well plates and screened for editing using Surveyor assay and sequencing of the *RNU12* locus.

### Immunoprecipitations

Anti-Sm (Y12; Invitrogen, MA5-13449) and anti-TMG (a gift from Dr Cindy Will, MPI Göttingen) antibodies were used for immunoprecipitation experiments. Following 48 h of siRNA transfection in 6-well plate format, HEK293 cells were scraped in 300 μl lysis buffer (20 mM HEPES, pH 7.4, 137 mM NaCl, 10% glycerol, 1% NP-40, 2 mM EDTA, 1× cOmplete protease inhibitor cocktail (Roche), 0.5 U/μl RiboLock) and sonicated 5 × 30 s using a Bioruptor Twin sonicator. Lysates were cleared by centrifugation (16 000 g, 15 min) and then incubated with antibodies (2 μg) bound to magnetic beads (Dynabeads Protein G) for 1 h at 4°C. Immunoprecipitation with anti-TMG antibody was carried out using 20 μg of Trizol-extracted total RNA in 20 mM HEPES, pH 7.4, 150 mM NaCl, 0.1% NP-40. After five washes with 200 μl lysis buffer, co-immunoprecipitated RNA was eluted from the beads by Proteinase K digestion and purified by phenol-chloroform extraction and ethanol precipitation.

### Cellular fractionation

Cellular fractionation was carried out using the REAP method as described (23). Total cell and cytoplasmic fractions were digested with proteinase K and extracted with phenol:chloroform:isoamyl alcohol and chloroform:isoamyl alcohol, followed by ethanol precipitation. RNA extraction from the nuclear pellet was carried out using TRIzol method.

### Northern blot

**Figure 2.**
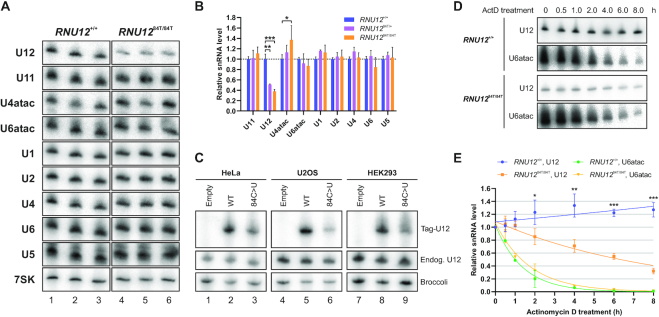
The 84C>U mutation leads to destabilization of the U12 snRNA. (**A**) Northern blot analysis of spliceosomal snRNA levels in *RNU12*^+/+^and *RNU12*^84T/84T^ cells. Shown are three independent samples from *RNU12*^+/+^ HEK293 cells (lanes 1–3) and samples from three different *RNU12*^84T/84T^ HEK293 single-cell clones (lanes 4–6). The same northern blot membrane was sequentially stripped and reprobed with different individual snRNA probes. For data visualization purposes, the signal intensity in the entire membrane (containing both *RNU12*^+/+^and *RNU12*^84T/84T^ samples) was adjusted so that the RNU12^+/+^ lanes showed similar intensities between the different snRNAs. (**B**) Quantification of snRNA northern blot data from *A*. The quantification is based on measurements from three total RNA samples from *RNU12*^+/+^ HEK293 cells, three samples from *RNU12*^84T/+^ HEK293 single-cell clones and eight samples from *RNU12*^84T/84T^ HEK293 single-cell clones. Loading was normalized by dividing all band intensities by the sum of U1 and U2 intensities averaged across all samples and the average intensity of the WT samples was then set to 1 for each snRNA. Error bars represent standard deviation. Statistical significance levels (ns: *P* > 0.05, [∗] *P* ≤ 0.05, [∗∗] *P* ≤ 0.01, [∗∗∗] *P* ≤ 0.001) from one-way ANOVA followed by Tukey's test are indicated. (**C**) Levels of exogenously expressed WT and 84C>U-mutant U12 snRNA in HeLa, U2OS and HEK293 cell lines. Tag-U12 constructs or empty pUC19 vector were co-transfected into each cell line together with a plasmid for expressing the F30-2xdBroccoli aptamer (61) used as a transfection control. (**D**) Actinomycin D treatment of *RNU12*^+/+^ and *RNU12*^84T/84T^ HEK293 cells. Cells were treated with 5 μg/ml actinomycin D for 0–8 h and harvested at the indicated time points. Shown are northern blots probed with U12 and U6atac snRNA-specific probes. (**E**) Decay kinetics of the U12 and U6atac snRNAs in *RNU12*^+/+^ and *RNU12*^84T/84T^ HEK293 cells upon actinomycin D treatment. The individual time series were normalized by setting the mean value of zero hour time point to 1.0. Statistical significance levels, determined using Student's *t*-test, refer to comparisons between U12_WT_ and U12_84C>U_ at the indicated time points.

Typically, 2–3 μg of Trizol-extracted total RNA was run on a 6–8% urea-polyacrylamide gel, transferred onto Hybond-XL nylon membrane using an Owl semi-dry or tank blotter and crosslinked with UV light using the UVP CL-1000 Ultraviolet Crosslinker. Membranes were probed either with 5 × 10^6^ cpm of [γ-^32^P]-ATP-labeled LNA/DNA or DNA oligonucleotides listed in Supplementary Table S1 or riboprobes produced by *in vitro* transcription with T7/T3/SP6 RNA polymerase in the presence of [α-^32^P]-UTP (major spliceosomal snRNAs in Figure 2A). Hybridization of DNA oligonucleotide probes was carried out overnight at 37 or 42°C (probes for major spliceosomal snRNAs and U5 snRNA) in 6xSSC, 25 mM Na_2_HPO_4_/NaH_2_PO_4_ (pH 7.4), 0.5% SDS, 5× Denhardt's solution, 150 μg/ml yeast RNA (Roche). For LNA-containing oligonucleotide probes and riboprobes, hybridization was carried out overnight at 45°C and 50% formamide was included in the hybridization buffer. Membranes probed with DNA probes were washed at room temperature in 2× SSC, 0.1% SDS and 0.5× SSC, 0.1% SDS, 15 min each. For LNA and RNA probes, two additional 15 min washes with 0.1× SSC, 0.1% SDS were included, the final wash done at 60°C. Blots were exposed on imaging plates and scanned using either Typhoon FLA 9400 or Fujifilm FLA-5100.

### Primer extension

Total RNA (20 μg) was ethanol precipitated with 1 pmol of ^32^P-labeled LNA primer (U12-RT-LNA-17) and annealed by heating to 95°C followed by slow cooling to 50°C. Annealing was carried out in 50 mM Tris–HCl (pH 8.3), 50 mM KCl, 1 mM EDTA. For extension, samples were adjusted to 50 mM Tris–HCl (pH 8.3), 75 mM KCl, 3 mM MgCl_2_, 10 mM DTT, 0.5 mM dNTPs, 50 μg/ml actinomycin D, 1 U/μl RiboLock, 2 U/μl Maxima H Minus RT. Extension reactions were incubated at 50°C for 30 min and terminated at 85°C for 5 min. After extension, template RNA was digested with RNase H and RNase A and the reactions were purified by phenol-chloroform (pH 7) extraction and ethanol precipitation. Extension products were analyzed on a 9% urea–polyacrylamide gel, which was dried, exposed on an imaging plate and scanned using Typhoon FLA 9400.

### RT-PCR

TRIzol-extracted RNA (1 μg) was treated with RQ1 RNase-Free DNase (Promega) to remove any contaminating genomic DNA. cDNA synthesis from 1 μg DNase-treated total RNA was carried out using Maxima H Minus Reverse Transcriptase (Thermo Fisher Scientific) and ReadyMade random hexamer primers (IDT) according to the manufacturer's instructions. PCRs were carried out using Phire Hot Start II DNA Polymerase (Thermo Fisher Scientific) and primers listed in Supplementary Table S1.

### Cloning and sequencing of U12 snRNA fragments

U12 snRNA was pulled down from 25 μg total RNA using 100 pmol of a biotinylated DNA oligo (hU12_1-23_B) and 60 μl of Dynabeads Streptavidin M270 beads. Beads were treated with RQ1 DNAse and then digested with Proteinase K to elute bound RNA. After removing beads, samples were extracted once with phenol:chloroform:isoamyl alcohol (25:24:1, pH 4.8) and ethanol precipitated. The S-RNA_cFP_loop oligo was pre-adenylated using 5′ DNA adenylation Kit (NEB). 5 pmol of the pre-adenylated oligo was then ligated to the 3′ ends of pulled down RNAs using 200 U of truncated T4 RNA Ligase 2 (NEB) at 16°C for 16 h in a 10 μl reaction volume. The enzyme was heat inactivated at 65°C for 5 min. The hairpin structure formed by the ligated DNA oligo was then used to prime cDNA synthesis, which was carried out using 200 U of Maxima H Minus RT (Thermo Scientific) in a 20 μl final volume at 50°C for 30 min, followed by heat inactivation at 85°C for 5 min. The RNA template was eliminated by digestion with RNase H. 5 pmol of annealed TruSeqP7/TruSeqP7c oligos were then ligated to the 3′ end of the cDNA using 30 U of T4 DNA ligase (Thermo Scientific) at 16°C for 16 h in a 30 μl reaction volume. The ligase was heat inactivated and the samples were treated with 1 U of USER enzyme (NEB) at 37°C for 30 min. 5 μl the cDNA was used for RT-PCR with primers U12-lig1 and U12-lig2. Samples were run on a 3.5% MetaPhor agarose gel, bands excised and DNA extracted. The purified PCR products were cloned using Zero Blunt TOPO PCR Cloning Kit (Thermo Fisher Scientific) and DNA from individual colonies analyzed by Sanger sequencing.

## RESULTS

### Construction of a cell line model of *RNU12*-mutant cerebellar ataxia

To study how the U12 snRNA mutation affects minor spliceosome function, we first introduced the 84C>T mutation into the genome of the HEK293 cell line using CRISPR/Cas9 genome editing. As C84 is located 19 nucleotides upstream of an AGG PAM site, we were able to utilize the Target-AID method (24) in which a specific guide RNA is used to target a fusion of Cas9 and activation-induced deaminase (AID) to the locus of interest. This enables specific deamination of cytosines within a narrow editing window centered at -18 relative to a PAM site (Figure 1B). We modified the system by replacing the G418 cassette in the original Target-AID plasmid with a puromycin cassette to allow for more efficient selection of transiently transfected cells. Analysis of the *RNU12* locus by mismatch cleavage assay and Sanger sequencing after transfection revealed efficient deamination at position –19 from the PAM site, corresponding to the 84C>T mutation (Figure 1B). Having confirmed editing at the transfected cell pool level, we carried out cloning by limiting dilution and screened the resulting clones for the presence of the mutation. We were readily able to obtain both heterozygous and homozygous 84C>T mutant cell lines, with 90% (45/50) of screened single-cell clones being positive for editing in the *RNU12* locus as judged by mismatch cleavage assay and ∼60% (11/18) of the positive clones analyzed by Sanger sequencing being homozygous for the 84C>T mutation. From here on, we will refer HEK293 cells carrying homozygous 84C>T mutation as *RNU12*^84T/84T^, heterozygous as *RNU12*^84T/+^ and wild-type cells as *RNU12*^+/+^. Similarly, the 84C>U-mutant U12 snRNA will be referred to as U12_84C>U_ and the wild-type snRNA as U12_WT_.

### The 84C>U mutation leads to reduced U12 snRNA stability in human cell lines

In the original report describing the 84C>T *RNU12* mutation (20), mononuclear blood cells from patients were reported to exhibit elevated levels of U12 snRNA compared to healthy wild-type individuals. Thus, we measured the levels of minor and major snRNAs in several homozygous and heterozygous HEK293 cell lines carrying the 84C>T mutation (Figure 2A, B). In contrast to the earlier report, our northern blot analyses revealed 49% and 62% reduction in steady-state U12 snRNA levels in *RNU12*^84T/+^ and *RNU12*^84T/84T^ clones, respectively. The reduced U12 snRNA levels with *RNU12*^84T/84T^ clones were also confirmed by primer extension analysis (Supplementary Figure S1). Additionally, the levels of the U4atac snRNA were slightly elevated in 75% (6/8) of the *RNU12*^84T/84T^ clones analyzed (*P* = 0.027; Figure 2A, B), reminiscent of the upregulation of U4atac in isolated growth hormone deficiency patients with *RNPC3* mutations (18). Levels of the other spliceosomal snRNAs were not significantly different from wild type HEK293 cells. Furthermore, we also confirmed that the *RNU12*^84T/84T^ mutation leads to aberrant splicing of U12-type introns, particularly activation of cryptic U2-type splice sites near the U12-type introns (Supplementary Figure S2), similarly as described earlier (25,26).

To test the generality of the observed reduction in U12 snRNA levels, we created a reporter construct in which the U12 snRNA is expressed from a genomic fragment that includes the native U12 promoter elements and 3′ flanking region (Supplementary Figure S3A). The 5′ end of the U12 snRNA carries a 15 nt sequence tag, allowing the exogenously expressed U12 snRNA to be distinguished from endogenous U12 snRNA by size on a northern blot or using tag-specific probe (Figure 2C, Supplementary Figure S3). The tag is predicted to remain single-stranded by RNAfold (27; Supplementary Figure S3B) and should thus not affect the folding of tagged U12 snRNA. We transfected WT and 84C>T-mutant versions of the tag-U12 constructs into HeLa (Figure 2C, lanes 1–3), U2OS (lanes 4–6) and HEK293 cells (lanes 7–9) and assayed tag-U12 snRNA levels by northern blot. Importantly, compared to the WT snRNA, exogenously expressed 84C>U-mutant U12 snRNA consistently exhibited reduced steady-state levels in all three cell lines tested (Figure 2C), confirming that the reduction in steady-state levels is not restricted to our edited HEK293 cell lines, and can be recapitulated using a reporter system.

Next, we asked whether the observed reduction in steady-state levels is due to reduced stability of the mutant snRNA, and treated *RNU12*^+/+^ and *RNU12*^84T/84T^ cells with the transcriptional inhibitor actinomycin D for 0–8 h and measured U12 snRNA levels at specific time points by northern blot (Figure 2D, E). Spliceosomal snRNAs have been reported to exhibit very long half-lives, with the notable exception of the U6atac snRNA, which turns over rapidly (28,29). Consistently, the wild-type U12 snRNA remained essentially stable for the duration of the experiment (8 h; Figure 2D, E). Compared to the WT snRNA, U12_84C>U_ displayed accelerated decay kinetics (*t*_1/2_ = 5.6 h), while the highly unstable U6atac snRNA exhibited similar half-lives in both wild-type and mutant HEK293 cells (*t*_1/2_ = 1.0 h and 1.2 h, respectively) that are consistent with a previous report (29). These results show that the 84C>U mutation has a destabilizing effect on the U12 snRNA.

### Truncated U12 snRNAs accumulate in 84C>T-mutant cells

**Figure 3.**
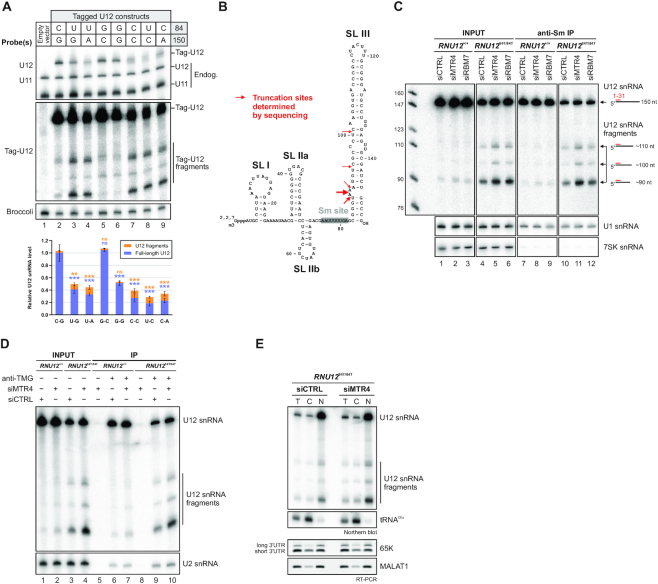
Identification and characterization of truncated U12 snRNAs in human cells. (**A**) Effects of mutations in the terminal base-pair of U12 snRNA stem-loop III on full-length U12 and U12 fragment levels. *Top:* Plasmid constructs for expressing tag-U12 with various combinations of nucleotides in the 84 and 150 positions were transfected into HeLa cells. Co-transfected F30-2xdBroccoli expression plasmid served as a transfection control. Total RNA from transfected HeLa cells was analyzed by northern blot with U12, U11, tag-U12 and Broccoli probes. *Bottom:* Quantification of full-length U12 (blue) and U12 fragment (orange) levels. Band intensities were normalized by the sum of all band intensities of each replicate, and the mean full-length U12 snRNA signal of WT tag-U12 (C-G) was then set to 1. Statistical significance was determined using one-way ANOVA followed by Dunnett's test. Significance levels above each bar refer to comparisons between WT tag-U12 and the indicated variant. Based on pair-wise comparisons (ANOVA with Tukey's test), differences in fragment levels between the group of variants showing low levels of tag-U12 fragments (C-G, G-C, G-G) and the group with high levels of fragments (U–G, U–A, C–C, U–C, C–A) were all statistically significant (*–***). In contrast, none of the pair-wise comparisons within the two groups showed statistically significant differences in fragment levels. (**B**) Location of truncation sites of U12 snRNA fragments determined by Sanger sequencing. See Supplementary Figure S4A and B for details. (**C**) Detection of short U12 snRNA species in association with Sm proteins. Forty eight hours after control, MTR4 or RBM7 siRNA transfection, anti-Sm immunoprecipitations were carried out using total cell lysates from *RNU12*^+/+^ and *RNU12*^84T/84T^ cells. RNA extracted from input and IP samples was analyzed by northern blotting with a probe specific to U12 (1–31). The U1 and 7SK snRNA were also analyzed as controls. The anti-Sm antibody immunoprecipitated the U1 snRNA, whereas the 7SK snRNA, which does not associate with Sm proteins, was only weakly detected. (**D**) U12 snRNA fragments carry a trimethylated 5′ cap. Anti-TMG immunoprecipitation was carried out using total RNA from *RNU12*^+/+^ and *RNU12*^84T/84T^ cells transfected with control or MTR4 siRNA. Northern blotting was carried out with U12 (1–31) and U2 snRNA (35–66) probes. (**E**) Cytoplasmic–nuclear distribution of U12 snRNA fragments. *RNU12*^84T/84T^ were fractionated into total (T), cytoplasmic (C) and nuclear (N) fractions and RNA from the fractions analyzed by northern blot and RT-PCR as indicated. tRNA(Glu) served as a marker for the cytoplasm, while the nuclear-retained lncRNA MALAT1 and the long-3′UTR isoform of the *RNPC3* mRNA (62) were used as nuclear markers.

To ask if weakening of the terminal base-pair of stem-loop III destabilizes the U12 snRNA, we tested a subset of nucleotide combinations at positions C84 and G150 using the 5′-tagged U12 snRNA expression construct and measured the steady-state levels of the tagged snRNAs in HeLa cells using northern analysis. Interestingly, our probes specific to the 5′ tag revealed, in addition to full-length U12 snRNA, additional shorter bands (Figure 3A, middle panel) that were more abundant with several of the mutations tested. Significantly, these bands were also detected in the *RNU12*^84T/84T^ cell lines with 5′-specific, but not 3′-specific probe. Consistently, cloning and sequence analysis revealed that the shorter RNA fragments represent U12 snRNAs that were truncated from the 3′-end (Figure 3B, Supplementary Figure S4A, B). These characteristics led us to hypothesize that the bands most likely represent stable decay intermediates arising from 3′-to-5′ exonucleolytic decay.

Mutational analysis revealed that any change that disrupts the base-pairing of SLIII terminal nucleotides reduce the full-length U12 snRNA levels by up to ∼80% (Figure 3A, lanes 7–9). Furthermore, the levels of the truncated U12 snRNAs are generally negatively correlated with the full-length U12 snRNA and account for a significant fraction (up to 41%) of the total cellular U12. Interestingly, while inversion of the terminal C–G base pair to G–C shows close to WT tag-U12 levels, a U–A base-pair at this position shows similar tag-U12 levels as the EOCA disease mutation leading to a U–G base-pair (n.s., ANOVA). Together these results suggest that the stability of the terminal base-pair of the SLIII is an important determinant of the overall stability of U12 snRNA. The only outlier is the noncanonical G–G pair that shows similar U12 snRNA levels as U–A and U–G pairs (n.s., ANOVA), but lacks the increase in U12 snRNA fragments. The reason for this is unknown, but a possible explanation may be that altered stacking interactions in the G-rich region of the lower stem may compensate for the loss of base-pairing interactions (see Figure 3B).

**Figure 4.**
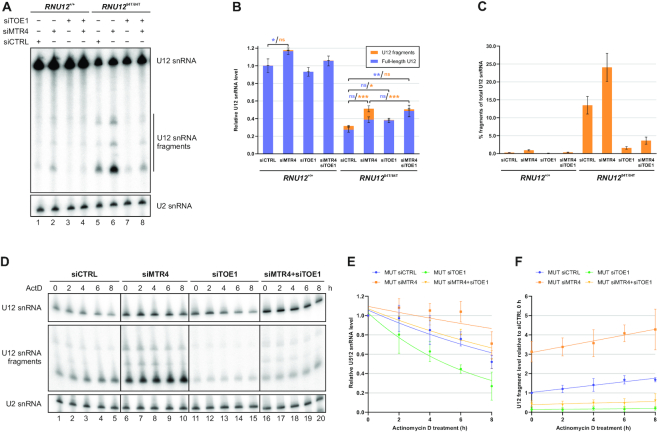
TOE1 is required for the formation of U12 snRNA fragments. (**A**) TOE1 plays a role in the formation or maintenance of U12 snRNA fragments. Total RNA from *RNU12*^+/+^ and *RNU12*^84T/84T^ cells transfected with control, MTR4, TOE1 or MTR4 and TOE1 siRNAs was analyzed by northern blot. (**B**) Effects of MTR4 and TOE1 knockdowns on full-length U12 snRNA levels (blue) and U12 snRNA fragment levels (orange). Quantification and normalization was carried out as described in Figure 3A. Statistical significance was determined by one-way ANOVA followed by Tukey's test. (**C**) Effects of MTR4 and TOE1 knockdowns on the percentage of U12 snRNA fragments of total U12 snRNA. (**D**) Impact of MTR4 and TOE1 knockdowns on the stability of the U12_84C>U_ snRNA and U12 snRNA fragments. 48 h after siRNA transfection, *RNU12*^84T/84T^ cells were treated with 5 μg/ml actinomycin D and harvested at the indicated time points. Total RNA was analyzed by northern blot using U12-specific probe binding to 5′ end of the snRNA. (**E**) Decay kinetics of the full-length U12_84C>_U snRNA in MTR4, TOE1 and MTR4/TOE1 double knockdown RNU12^84T/84T^ cells. Quantification of northern blot data from three independent experiments represented in panel D. Full-length U12 snRNA signal was normalized to the U2 snRNA signal and the 0 h sample of each set of samples was set to 1. (**F**) Time-dependent accumulation of U12 snRNA fragments during transcriptional inhibition. The quantification is based on the highest abundance (∼90 nt) U12 snRNA fragments only and the data is derived from three independent experiments. Loading was normalized by U2 snRNA signal. For each replicate, the 0 h siCTRL sample was set to 1 to visualize both accumulation and absolute differences in U12 snRNA fragment levels between siRNA treatments.

The truncated species are also present in HEK293 cells carrying WT U12 allele. In that case the truncated forms accounted for only a minute fraction (∼0.3%) of the total U12 snRNA as compared to the ∼13.5% with 84C>T mutation (Figure 3C). In both cases, knockdown of the NEXT complex (see below) significantly increased the levels of the decay intermediates. The U12 snRNA fragments contain the Sm site but are missing most of the stem-loop III sequence (Figure 3B). This is in contrast to previously described truncated forms of the U1 and U2 snRNAs, which lack the Sm site (30,31). Unlike truncated forms of U1 snRNA, which were shown to be highly unstable (30), the most abundant U12 snRNA fragments of ∼90 nt remained stable for 8 h of actinomycin D treatment (see figures 4D and F); however, the lower-abundance fragments appeared to decay faster, although their very low levels made half-life estimation unfeasible. We conclude that the decay of the mutant U12 snRNAs utilizes a natural pathway also acting on WT U12 snRNA, as the truncated decay intermediates can also be detected in WT HEK293 cells. However, in *RNU12*^84T/84T^ cells a significant fraction of U12 snRNA is targeted via this pathway, thus lowering the steady-state levels of U12 snRNA.

### U12 snRNA fragments are formed after cytoplasmic snRNP biogenesis steps

We next investigated whether the 84C>U mutation affects, in addition to the accelerated decay, also the overall U12 snRNP biogenesis. snRNPs form through a complex biogenesis pathway that is distinct for Sm-class and LSm-class snRNPs (reviewed in 32,33). Sm-class snRNAs, which include all spliceosomal snRNAs except U6 and U6atac, are transcribed by RNA polymerase II and cleaved by the Integrator complex to generate precursor snRNAs with a 3′ extension (34,35), which are subsequently exported to the cytoplasm for further biogenesis steps. These include assembly of the Sm ring on the Sm site of the snRNA and hypermethylation of the 7-methylguanosine cap (m^7^G) to 2,2,7-trimethylguanosine (m^2,2,7^G or TMG). We asked whether the mutant U12 snRNA and the U12 fragments have successfully undergone the cytoplasmic steps of snRNP biogenesis by immunoprecipitation with anti-Sm (Y12) and anti-2,2,7-trimethylguanosine cap (anti-TMG) antibodies. We found that both the full-length WT and 84C>U-mutant snRNAs and their truncated forms were all efficiently immunoprecipitated, not only with the anti-Sm antibody (Figure 3C, lanes 7–12) but also with anti-TMG antibody (Figure 3D). This suggests that full-length U12 snRNA and the truncated forms successfully complete both the Sm core assembly and the subsequent 5′ cap hypermethylation steps of the snRNP assembly that take place in cytoplasm (36,37). Consistent with the role of Sm core and TMG cap as a bipartite signal for nuclear import of snRNPs (38,39), nucleocytoplasmic fractionation of *RNU12*^84T/84T^ cells revealed that U12 snRNA fragments are predominantly located in the nucleus, with only a minor sub-population detected in the cytoplasm (Figure 3E).

The prime candidate mediating U12 snRNA decay is the nuclear exosome targeting (NEXT) complex. This trimeric cofactor complex consists of MTR4, RBM7 and ZCCHC8 proteins (40) and has been shown to target snRNAs for nuclear exosome-mediated decay (41). To test if the NEXT complex is involved in the decay of 84C>U U12 snRNA, we knocked down MTR4 and RBM7 in *RNU12*^+/+^ and *RNU12*^84T/84T^ cells (Supplementary Figure S5). Northern analysis revealed a mild upregulation of the full-length U12 snRNA with MTR4 and RBM7 knockdowns in *RNU12*^+/+^ cells (1.17-fold increase, *P* = 0.016) while the result in *RNU12*^84T/84T^ cells was not conclusive due to elevated variation (1.4-fold, n.s.) (Figure 4A, B). In contrast, the levels of U12 snRNA fragments increased significantly both in total RNA pool (Figure 3C, lanes 1–6) and anti-Sm immunoprecipitates (Figure 3C, lanes 7–12) after MTR4 or RBM7 knockdown, and also in anti-TMG immunoprecipitates after MTR4 knockdown (Figure 3D, lanes 5–10). Compared to non-targeting siRNA control, knockdown of MTR4 resulted in a 4-fold (n.s.) and 3-fold (*P* <0.0001) increase in fragment levels in *RNU12*^+/+^ and *RNU12*^84T/84T^ cells, respectively (Figure 4A, lanes 1, 2, 5, 6; Figure 4B). The percentage of U12 fragments increased from 0.3% to 0.9% in *RNU12*^+/+^ cells, while in *RNU12*^84T/84T^ cells the percentage increased from 13.5% to 24.1% (Figure 4C). This data suggested that the NEXT complex is involved in targeting the U12 snRNA fragments for exosome-mediated decay.

Recent work revealed TOE1, a primarily Cajal body-localized deadenylase with nucleo-cytoplasmic shuttling activity (42,43), as the enzyme responsible for the 3′ trimming of human snRNA precursors to their mature length (44,45). TOE1 thus represents another 3′-to-5′ exonucleolytic activity targeting the 3′ end of snRNAs. Somewhat surprisingly and in contrast to NEXT complex knockdowns, TOE1 knockdown led to a decrease in the levels of the U12 snRNA fragments in both WT and mutant HEK293 cells (Figure 4A–C). In *RNU12*^84T/84T^ cells TOE1 knockdown resulted in a 6.9-fold decrease in U12 fragment levels (*P* = 0.049), reducing the percentage of fragments from 13.5% to 1.6%. Similarly, TOE1 knockdown in *RNU12*^+/+^ cells led to almost complete disappearance of the U12 fragments (Figure 4A, C). Combining siMTR4 with siTOE1 resulted in slightly elevated U12 fragment levels than with siTOE1 alone, but well below siCTRL levels, while full-length U12 levels in *RNU12*^84T/84T^ cells showed a significant (*P* = 0.0017) 1.8-fold increase compared to siCTRL (Figures 4A, lane 8; B). The results with U12 snRNA fragments suggest that the decay of the mutated U12 snRNA is initiated by TOE1, which forms the 3′-truncated U12 snRNA fragments that are subsequently targeted for further decay by the NEXT complex.

Half-life measurements (Figures 4D–F) provide further insight to the role of TOE1 and NEXT complex in the decay of the U12_84C>U_ snRNA. Knockdown of MTR4 led to initial stabilization not only of the full-length U12_84C>U_ snRNA but also U12 snRNA fragments, suggesting that the nuclear exosome, targeted by the NEXT complex, has a significant role in the decay of full-length U12_84C>U_ snRNA (Figure 4D, E), but also in the final decay of the U12 snRNA fragments (Figure 4D, F). In contrast, the reduced half-life of full-length U12_84C>U_ snRNA observed with TOE1 knockdown that is restored to control knockdown levels in double-knockdown of TOE1 and MTR4 (Figure 4D, E) suggests that TOE1 has also a role in counteracting the exosome-dependent decay. We do note that since the combined NEXT complex and TOE1 knockdowns (Figure 4B) had only a minor effect on restoring the levels of full-length U12_84C>U_ snRNA, other RNA decay pathways are likely to play a role in the decay of U12_84C>U_. Indeed, several pathways have been implicated in the quality control and normal turnover of snRNAs (46–50).

### SLIII point mutations destabilize U12 snRNA

**Figure 5.**
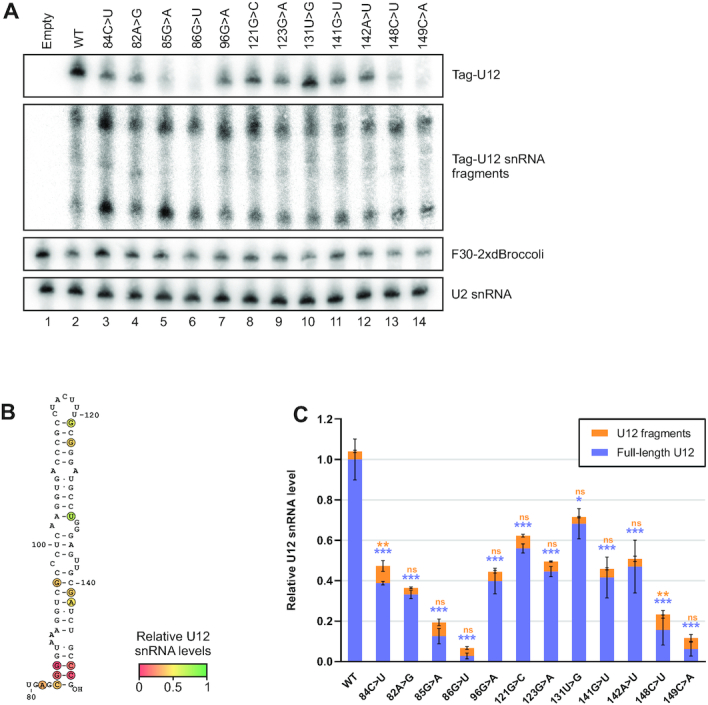
Effect of stem-loop III variants on U12 snRNA levels. (**A**) Effects of single-nucleotide variants in the stem-loop III of U12 snRNA on full-length tag-U12 and tag-U12 fragment levels. (**B**) Impact of the analyzed variants on tag-U12 levels in relation to location on the stem-loop III. The color scale indicates relative tag-U12 levels, with WT tag-U12 level set to 1 (green), and 0 (red) indicating complete loss of tag-U12 signal. (**C**) Effect of stem-loop III variants on full-length tag-U12 levels (blue) and tag-U12 fragment levels (orange). Quantification and statistical testing were carried out as described for Figure 3A.

The dbSNP and gnomAD databases (51,52) list a significant number of SNP and other variants in U12 snRNA including the SLIII region (Supplementary Table S2). Considering the magnitude of destabilization induced by single-nucleotide changes in the very stable stem-loop III (Figure 3A), we reasoned that other U12 snRNA alleles segregating in human population, particularly those with nucleotide substitutions in the stem–loop III may have a similar effect on U12 snRNA stability. These could include, in addition to SNPs directly affecting the base of SLIII, also other variants that change the folding of SLIII and potentially result in targeting by TOE1 and/or NEXT complex. To test this possibility, we sampled a subset of the dbSNP/gnomAD mutations and other mutations (Figure 5B) predicted to perturb the structure of the snRNA (Supplementary Table S3). For predictions we used the RNA2DMut tool (53) that estimates the effect of all possible point mutations on the secondary structure of an input RNA, providing a statistical metric describing the structural ensemble (ED, ensemble diversity) as well as minimum free energy and centroid secondary structures. Using the tag-U12 reporter, we analyzed a panel of mutations in the 3′ domain of U12 snRNA which all showed elevated ED values compared to the WT structure (ΔED), indicating presence of multiple structural conformations or lack of structure (Figure 5).

Interestingly, all the analyzed variants showed lower steady-state tag-U12 levels compared to the WT tag-U12 snRNA (Figure 5), regardless of whether they were located at the base of SLIII or elsewhere. In particular, mutations in the G85–C149 (85G>A, 149C>A) and G86–C148 (86G>U, 148C>U) base pairs at the base of SLIII had a very dramatic effect, leading to 84–97% reduction in the full-length tag-U12 signal (Figure 5A, lanes 5, 6, 13, 14; Figure 5C). Furthermore, several other mutations located further up in the SLIII stem showed similarly reduced U12 snRNA levels as the original 84C>U mutation. In addition to the 84C>U mutation, a significant increase in tag-U12 fragment levels was only observed with the 148C>U variant. This data indicates that in addition to 84C>U, multiple additional mutations in the SLIII, a subset of which are already segregating in human population, can destabilize U12 snRNA and are thus alone or as compound heterozygotes a potential cause of additional minor spliceosome-associated human diseases.

## DISCUSSION

**Figure 6.**
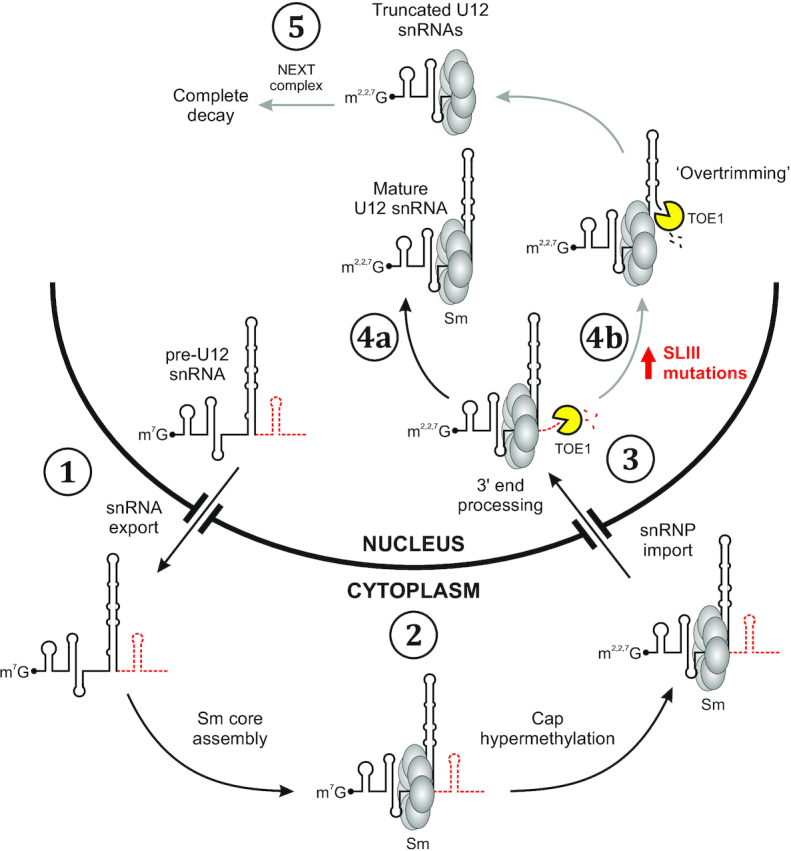
A model for U12 snRNA quality control/decay pathway. U12_WT_ and U12_84C>U_ snRNAs share the early steps of the snRNP biogenesis (Steps 1–3) that include transcription/early processing, cytoplasmic snRNP biogenesis steps, and nuclear import of the core snRNP. Subsequently, the U12_WT_ snRNA undergoes 3′ end trimming by TOE1 (Step 4a), giving rise to mature U12 snRNP. With U12_WT_ the trimming with TOE1 can infrequently progress too far (‘overtrimming’), causing low-level formation of truncated U12 snRNAs in WT cells (Step 4b) that are further degraded by the NEXT complex-dependent exonucleolytic process (Step 5). Mutations in the SLIII, such as U12_84C>U_, lead to increased utilization of the overtrimming pathway, leading to accumulation of truncated U12 snRNAs and decreased levels of full-length U12 snRNA. The U12 precursor structure, with extension indicated with red dotted line has been drawn according to Yong *et al.* (63).

In this study, we have investigated the molecular mechanism underlying Early onset cerebellar ataxia caused by the 84C>T mutation in the *RNU12* gene encoding U12 snRNA, a component of the minor spliceosome. We show that the *RNU12* 84C>T mutation leads to a significant reduction in the half-life of U12 snRNA and consequently ∼60% decrease in the steady-state levels of the U12 snRNA not only in a HEK293 cell model carrying homozygous 84C>T mutation, but also in several other cell lines. Additionally, we discovered that HEK293 cells carrying the mutated U12 snRNA accumulate stable decay intermediates that lack the 3′ SLIII of the U12 snRNA. Furthermore, we show that efficient decay of the U12_84C>U_ snRNA requires TOE1 deadenylase and NEXT complex activities. Given that both the full-length U12_84C>U_ snRNA and the decay intermediates carry a TMG cap and are associated with Sm proteins, we propose a model (Figure 6) where the decay of the U12_84C>U_ snRNA takes place in the nucleus after the cytoplasmic steps of the snRNA biogenesis have been completed. This decay/quality control pathway operates at low levels in WT cells, but the mutations that compromise the integrity of the SLIII shift the balance towards the decay pathway.

Our observation that the 84C>T mutation leads to a reduction in the half-life and consequently reduced steady-state levels of the U12 snRNA in multiple cell lines is in contrast to the finding by Elsaid *et al.* (20) who used qRT-PCR analysis of patient mononuclear blood lymphocytes, and instead reported upregulation in the U12_84C>U_ snRNA levels. Additionally, while we also detect minor-intron specific splicing defects, those are less prominent than the ones described in Elsaid *et al.* (20) work. Such strong tissue and cell type-specific effects on intron retention levels have also been described with MOPD1/TALS patient cells containing mutations in U4atac snRNA (54). In contrast, the contradicting outcome on U12_84C>U_ snRNA levels between the two studies either suggest differences between the proliferating cells in culture and patient-derived peripheral blood lymphocytes or, alternatively, technical differences between the two studies. The U12 snRNA downregulation in this study was observed by two different methods (northern analysis using multiple probes and primer extension analysis). Furthermore, the NEXT complex and TOE1-dependent destabilization of the U12_84C>U_ snRNA, observed in this study, is consistent with the recent work on snRNA decay pathways (45). The 2-fold increase in U12_84C>U_ snRNA levels observed in Elsaid *et al.* (20) in lymphocytes would require either increased stability or transcriptional upregulation of the U12_84C>U_ snRNA. However, the location of the 84C>T mutation outside of the known promoter region (55,56) argues against the possibility that the mutation could lead to transcriptional upregulation. Similarly, the already high stability of U12_WT_ snRNA (29; Figure 2D, E) questions whether the upregulation reported by Elsaid *et al.* (20) is achievable by further increases in stability, unless there are lymphocyte-specific biological mechanisms for upregulation that are also shared with the cells of cerebellar tissue.

The observation that U12_84C>U_ snRNA is specifically targeted for 3′-to-5′ exonucleolytic decay by TOE1 and the NEXT complex generating relatively stable decay intermediates that terminate downstream of the Sm-site provide an insight to the mechanism of the decay pathway. The kinetics of accumulation of the shortened U12_84C>U_ snRNA fragments after transcription inhibition (Figure 4F) suggests that the 3′ end decay takes place only after nuclear reimport instead of the cytoplasmic phase of snRNA biogenesis or immediately after transcription. This conclusion is consistent with the localization of NEXT complex in nucleoplasm (40) and TOE1 in Cajal bodies (42,43), and further supported by the presence of Sm core proteins and TMG cap in the U12_84C>U_ snRNA fragments, as these jointly constitute the nuclear import signal for core snRNPs (38,39). In contrast, defects in the early stages of the snRNA biogenesis are instead known to lead to a loss of cap hypermethylation and inhibition of nuclear reimport and subsequent rapid decay in cytoplasmic P-bodies, as observed with truncated forms of U1 and U2 snRNAs (U1-tfs and U2-tfs, respectively) that have errors in 3′ end formation and lack the Sm-site (30,31).

TOE1 and the NEXT complex knockdown data further suggests that the process is initiated by TOE1 which can, particularly with the U12_84C>U_ snRNA, ‘overtrim’ the 3′ end of the snRNA and lead to the formation of truncated U12 snRNA fragments. The formation of discrete size U12 snRNA fragments that are visible in northern analysis most likely reflects the inability of TOE1 to dislocate the Sm proteins from the Sm site. This model is consistent with the role of Sm proteins in protecting snRNA from degradation (46,57) and is also supported by earlier RNase footprinting experiments showing that the protective effect of Sm proteins on U12 snRNA extends from the Sm site to the beginning of SLIII (nucleotides 69–95) (58). To overcome this protective barrier, subsequent action by nuclear exosome targeted by the NEXT complex is required for further degradation of the U12 fragments.

What is the mechanistic basis of the U12_84C>U_ destabilization? The low levels of U12 snRNA decay intermediates detected also in the WT cells argue that the accelerated decay of U12_84C>U_ snRNA is part of normal turnover process. We hypothesize that with WT U12 snRNA, an infrequent stochastic process of terminal base-pair melting (59) at the base of SLIII can expose an unpaired 3′ nucleotide and provide an entry point for the exonuclease activities. Based on our mutagenesis studies (Figure 3A) we suggest that mutation of the terminal C-G base pair to U-G base-pair at the base of SLIII exacerbates this melting process, and thus provides an opening for TOE1 action. Supporting this hypothesis, the importance of the integrity of the lower stem of SLIII on minor spliceosome function has also been reported in an earlier mutagenesis study by Sikand and Shukla (60).

In addition to the mutations that directly affect the stability of lower stem of SLIII, other mutations elsewhere in SLIII may also affect the stability through inducing more extensive structural changes in SLIII. This possibility is supported by our analysis of additional low-frequency U12 snRNA allelic variants with mutations in SLIII that segregate in human population. A subset of those were predicted by the RNA2DMut tool to increase the ensemble diversity of the U12 snRNA, suggesting a possible SLIII folding heterogeneity that could also lead to the observed reduction in the U12 snRNA steady-state levels. Given that several of the analyzed SNP variants reduce U12 snRNA steady-state levels to the same level as the 84C>T mutation, or even below, our data thus support the possibility for additional disease-causing variants within the *RNU12* locus.

The role of TOE1 in initiating the U12_84C>U_ decay is consistent with recent studies (44,45) that have linked TOE1 not only to snRNA maturation, but also quality control. Specifically, a recent work by Lardelli and Lykke-Andersen (45) has indicated that TOE1 and the NEXT complex can play an antagonist role in snRNA decay, where TOE1 shields the snRNA 3′ end from the nuclear exosome. Such competition between the two activities is consistent with our half-life analyses (Figure 4D, E) where the MTR4 knockdown stabilizes and TOE1 knockdown destabilizes the full-length U12_84C>U_ snRNA. It is possible that the role of TOE1 in initiating the decay of U12_84C>U_ snRNA is also linked to the tissue specificity of the disease. Significantly, mutations in *TOE1* have been shown to cause pontocerebellar hypoplasia (PCH7; 44) which, together with the EOCA mutation in this study, suggests that specific cell type(s) in cerebellum may have increased demand for TOE1 and other snRNA 3′ end processing activities that could make U12_84C>U_ more vulnerable for degradation. Alternatively or additionally, as a variety of neuronal phenotypes is shared among the minor spliceosome diseases (12), particularly MOPDI/TALS, RFMN and LWS with U4atac snRNA mutations, the cerebellar defect may rather be arising from splicing defects in a specific set of genes carrying minor introns.

## Supplementary Material

gkab048_Supplemental_Files
