# Supplementary material for: The integrity of the U12 snRNA 3′ stem–loop is necessary for its overall stability
Source: Nucleic Acids Res. 2021 Feb 12;49(5):2835–47. doi: 10.1093/nar/gkab048 (PMC7968993; doi:10.1093/nar/gkab048)
Supplement: gkab048_Supplemental_Files [file gkab048_supplemental_files.zip › SupplementaryMaterial.pdf]

Supplementary material for

The integrity of the U12 snRNA 3' stem-loop is necessary for its overall stability

Antto J. Norppa, Mikko J. Frilander

Supplementary Figure S1

Supplementary Figure S2

Supplementary Figure S3

Supplementary Figure S4

Supplementary Figure S5

Supplementary Table S1

Supplementary Table S2 (provided as a separate file)

Supplementary Table S3 (provided as a separate file)

Supplementary Figure S1

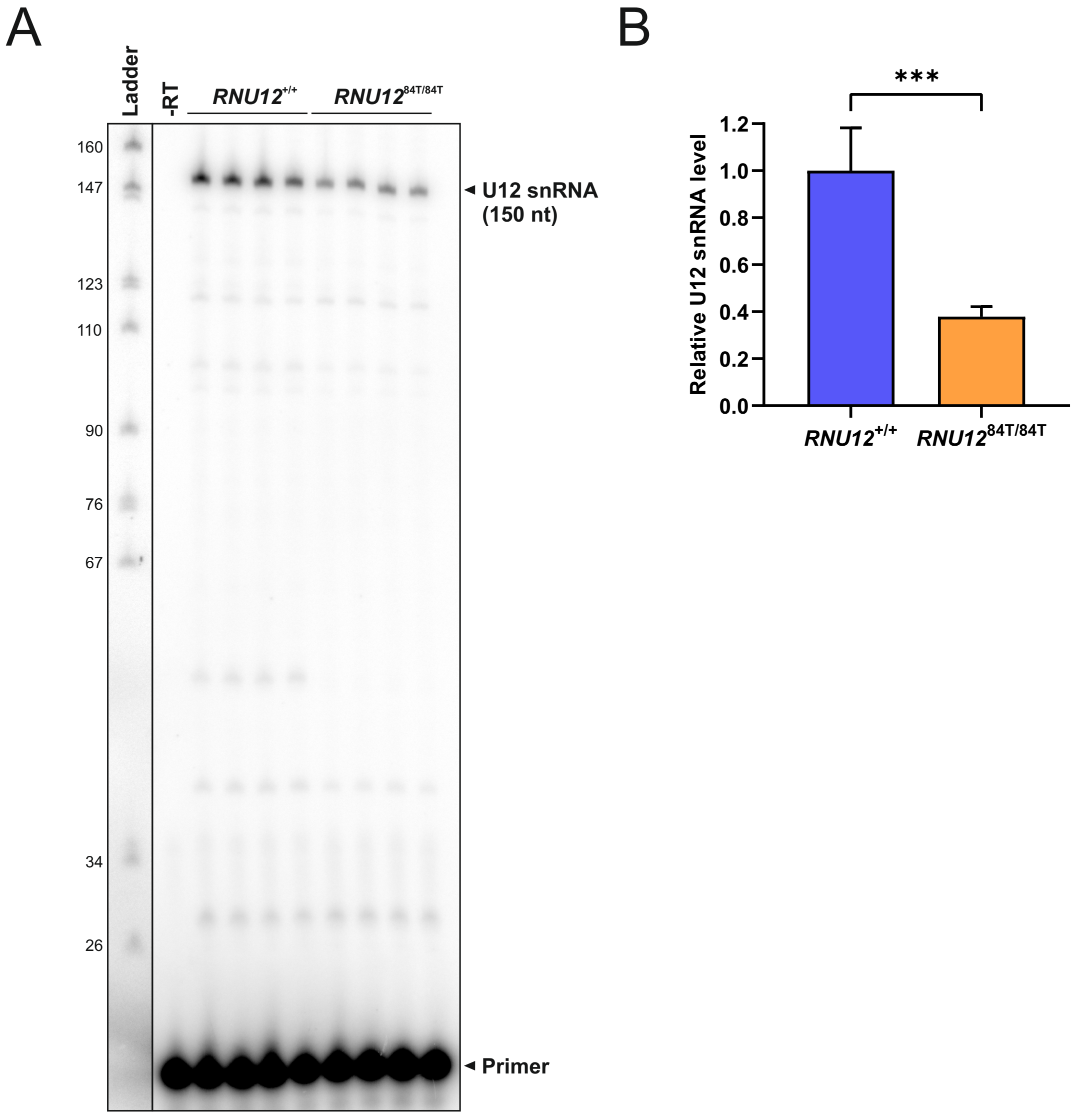

Figure S1. U12 snRNA levels in *RNU12*$^{+/+}$ and *RNU12*$^{84T/84T}$ cells analyzed by primer extension. **(A)** Total RNA from four separate cultures of *RNU12*$^{+/+}$ and four individual *RNU12*$^{84T/84T}$ single-cell clones was subjected to primer extension using a DNA/LNA primer specific to U12 nucleotides 134–150 . **(B)** Quantification of the gel shown in *A*. For each sample, U12 intensities were normalized to primer intensities, and the average normalized U12 intensity of *RNU12*$^{+/+}$ samples was then set to 1. Unpaired *t*-test, p=0.0058.

Supplementary Figure S2

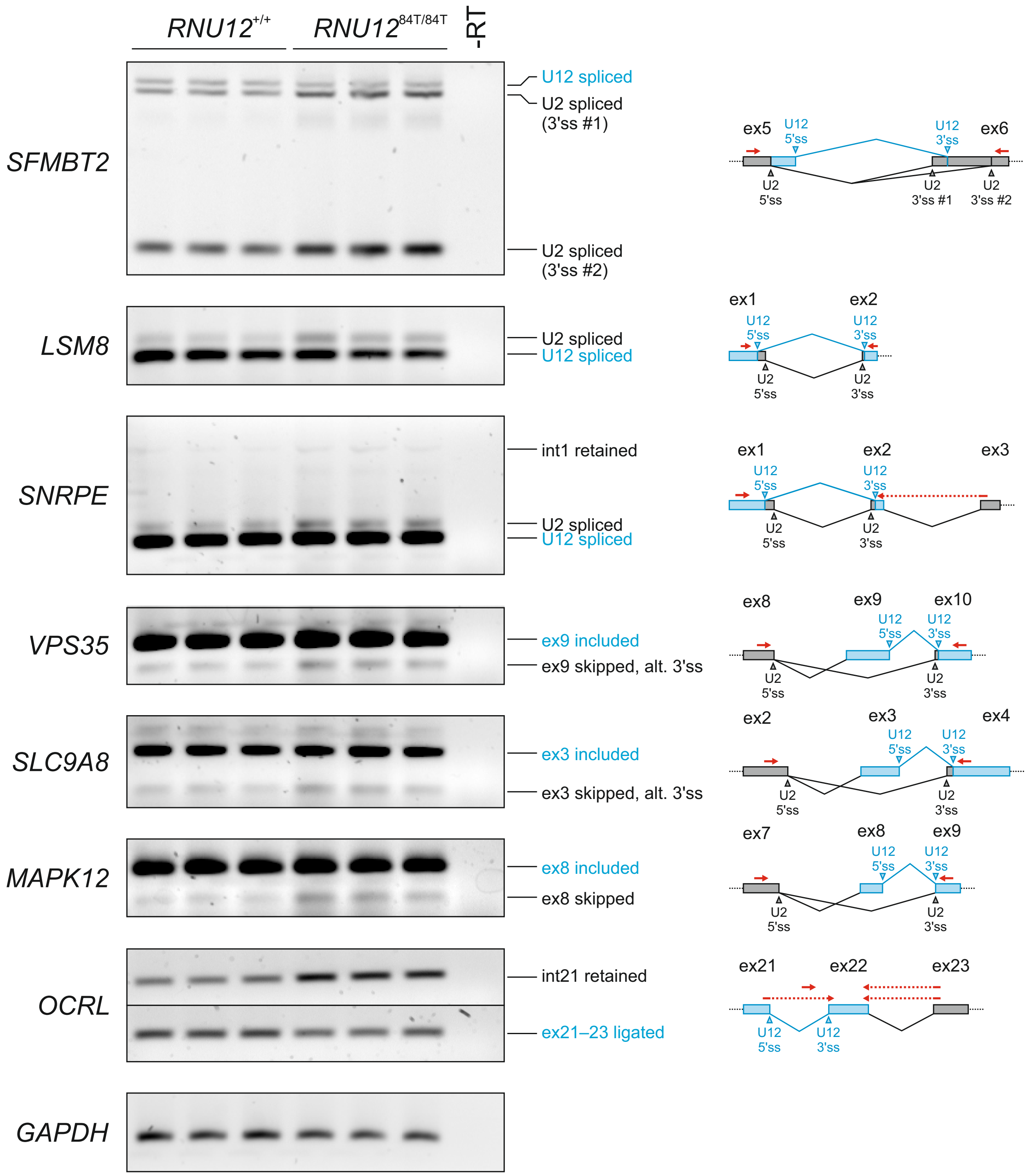

Figure S2. Aberrant splicing of U12-type introns in *RNU12*$^{84T/84T}$ cells. *Left:* RT-PCR splicing analyses using random primed cDNA from three independent cultures of *RNU12*$^{+/+}$ cells and three monoclonal *RNU12*$^{84T/84T}$ cell lines. Primers used are listed in Supplementary Table S1. *Right:* Schematics showing the observed splicing patterns, with splicing products utilizing U12-type splice sites highlighted in blue. Location of primers is indicated as red arrows. *SFMBT2: RNU12*$^{84T/84T}$ cells show increased usage of a U2-type 5' splice site (5'ss) and two alternative 3' splice sites (3'ss) in the vicinity of the U12-type intron. *LSM8, SNRPE:* Usage of cryptic U2-type 5'ss and 3'ss inside the U12-type intron is increased in *RNU12*$^{84T/84T}$ cells. *VPS35*, *SLC9A8*: Increased exon skipping, with utilization of an alternative 3'ss, is observed in *RNU12*$^{84T/84T}$ cells. *MAPK12:* Skipping of exon 8 is elevated in *RNU12*$^{84T/84T}$ samples. *OCRL:* Retention of the U12-type intron (intron 21) is increased, and a concomitant decrease in exon 21–23 ligated product is observed in *RNU12*$^{84T/84T}$ cells. *GAPDH:* loading control.

Supplementary Figure S3

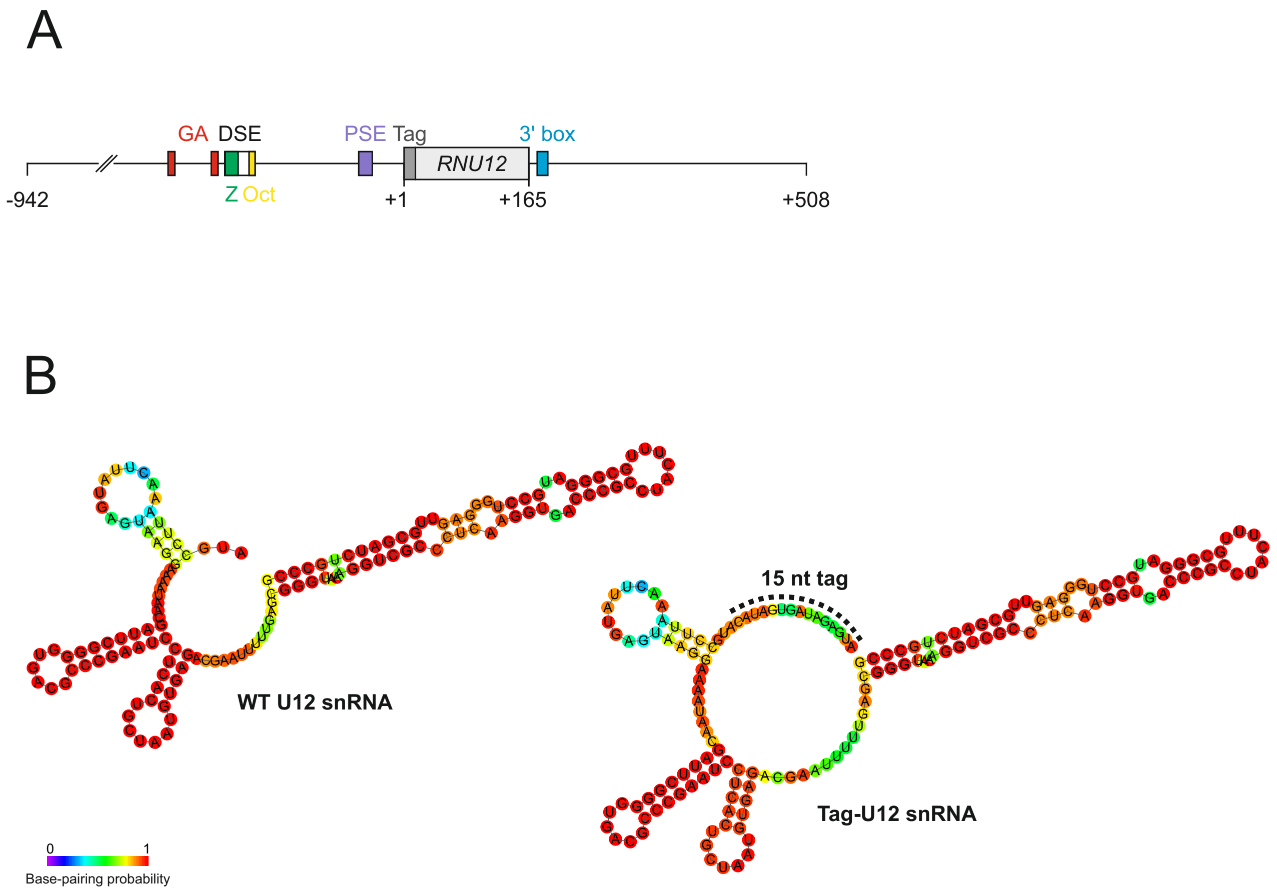

Figure S3. (A) Schematic of the U12 snRNA expression construct used to analyze the effect of variants on U12 snRNA levels. Locations of the snRNA promoter and 3' processing elements shown are according to Faresse et al. (2012). These are the PSE, proximal sequence element (violet); the DSE, distal sequence element, composed of a Z-motif (green) and an octamer motif (yellow); GA motifs (red); and the 3' box (cyan). **(B)** RNAfold web server prediction of the secondary structure of wild-type U12 snRNA (left) and U12 snRNA with the inserted 15 nt tag sequence (right)

Supplementary Figure S4

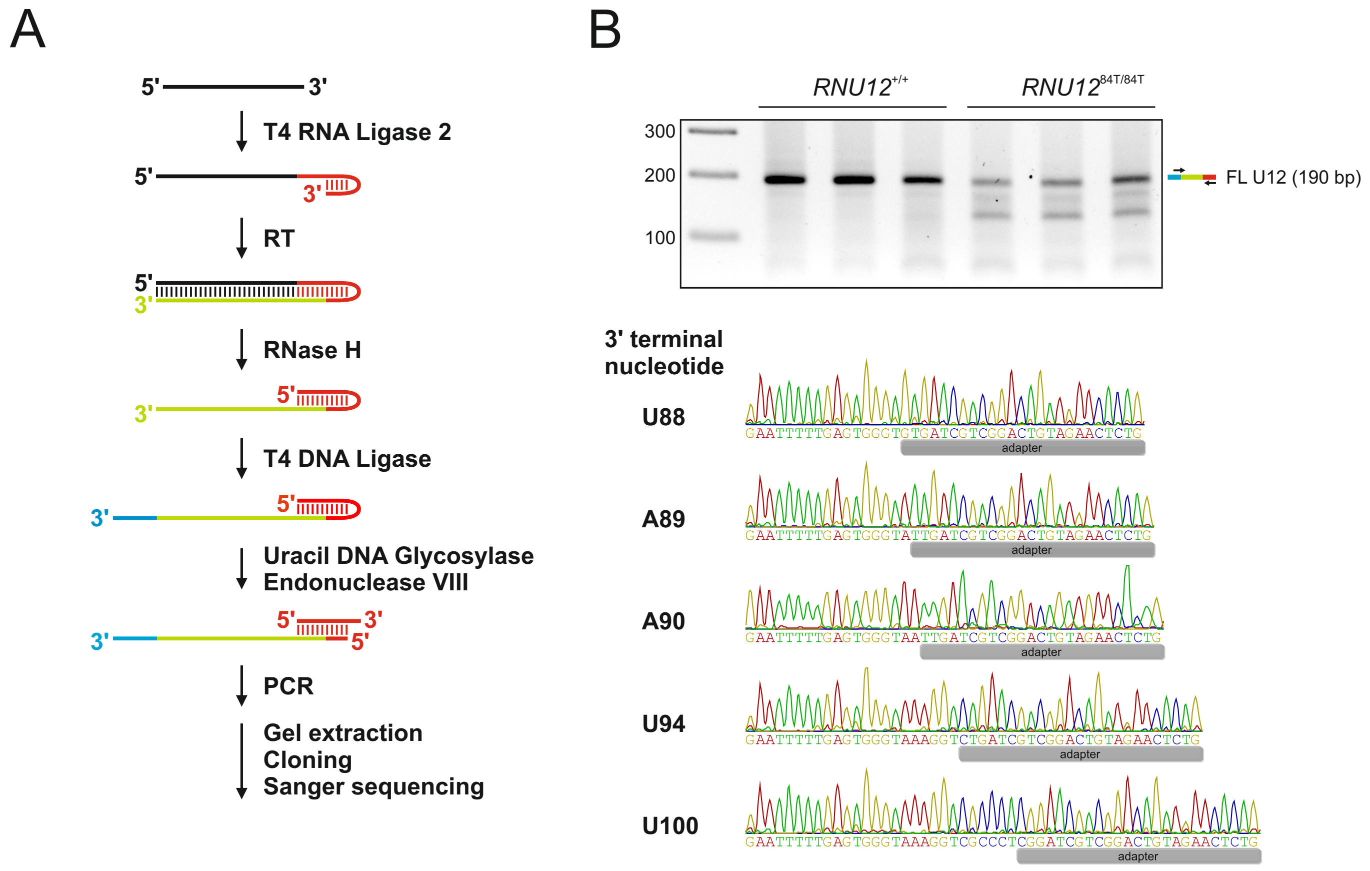

Figure S4. (A) Procedure used for sequencing the U12 snRNA fragments. We used a 5'-end-specific biotinylated oligo and streptavidin beads to capture U12 snRNA from total RNA of WT and 84C>T-mutant HEK293 cells. We then ligated an adapter to the 3' end of the pulled down RNAs and carried out cDNA synthesis and ligation of a second adapter to the 3' end of the cDNA. **(B)** RT-PCR with adapter-specific primers using cDNA prepared as depicted in *A*. Consistent with northern blot analysis, RT-PCR using adapter-specific primers revealed reduced levels of the full-length U12 snRNA but prominent increase in the levels of shorter species in the mutant cells. Gel extraction followed by cloning and Sanger sequencing of these species identified them as U12 snRNA fragments truncated at the 3' end. The most abundant of the truncated forms terminated at position 88–90, with the most common 3'-terminal nucleotide being A89, while few sequences were also obtained that terminated further downstream (positions 94 and 100).

Supplementary Figure S5

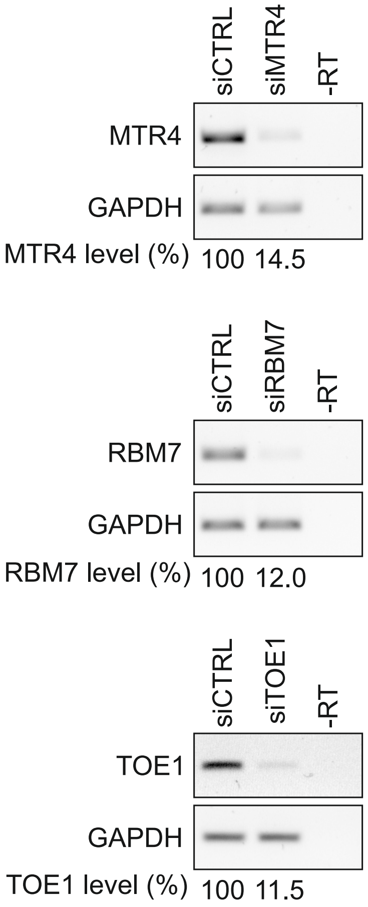

Figure S5. Knockdown efficiency of siRNAs used in this study. MTR4, RBM7 and TOE1 mRNA levels were analyzed by RT-PCR 48 h after siRNA transfection. mRNA levels relative to non-targeting control siRNA (siCTRL) are indicated below each gel. siRNA sequences are listed in Supplementary Table S1.

Supplementary Table S1. Oligonucleotides used in this study

PCR primers

Name	Sequence	Purpose
RNU12-BamHI-F	cctaggatccAGAGGCGAGTGTTAACAGGG	Cloning RNU12 gene and flanking regions to the pUC19 vector
RNU12-HindIII-R	tgataagcttGAACTGAGCTGCTGTGTGAG	
U12-5'tag-F	gtgatacATGCCTTAAACTTATGAGTAAG	Site-directed mutagenesis to create pUC19-RNU12-5'tag
U12-5'tag-R	tatctcatAAGGACGTTTTTAGTTTATTTC	
U12-surv-F	CTAGGGAGCGACGAACTACT	Amplifying the RNU12 locus for Surveyor assay and sequencing
U12-surv-R	CCAACGTCAATACTGTGGGC	
MALAT1-1	GTTGGCACGAACACCTTCAG	RT-PCR primers for MALAT1 lncRNA
MALAT1-2	TGGCCTACTCAAGCTCTTCT	
h65K-79	GGGGGAAGGACAAACATTTC	RT-PCR primers for RNPC3 long and short-3'-UTR isoforms
h65K-88	CCAGGTGGTCAATTTCTTCC	
h65K-196	GCTCGATCTGCTAGACCAAAA	
MTR4-1	TGGAAAGAAGCCCAGGATAGA	RT-PCR primers for MTR4 mRNA
MTR4-2	ACGGGTATTCCTTAGCAGCT	
RBM7-1	TCAGAGACAAGCAGTGATGAAC	RT-PCR primers for RBM7 mRNA
RBM7-2	CTGTTCCCGGCTATAATGTCT	
TOE1-1	TGCCTTCCGGAAATGTGAAC	RT-PCR primers for TOE1 mRNA
TOE1-2	AGCCGAGAAGTTGTCACAGA	
hGAPDH-1	CACCAGGGCTGCTTTTAACT	RT-PCR primers for GAPDH mRNA
hGAPDH-2	TGGAAGATGGTGATGGGATT	
SFMBT2-ex5-F	CACAGACTGGACAGAATTTCTCA	RT-PCR primers for alternative 5'ss / 3'ss usage within SFMBT2 ex5 / ex6
SFMBT2-ex6-R	TCTCTTGACACCAACCAACTG	
LSM8-1	TTCAGTTCTGCTTGCTGTCG	RT-PCR primers for alternative 5'ss / 3'ss usage within LSM8 intron 1
LSM8-3	CCCATCTGATGTAATAACGGCA	
SNRPE-ex1-F	TCCGGAAGTTGCTCTCAGAG	RT-PCR primers for for alternative 5'ss / 3'ss usage within SNRPE intron 1
SNRPE-ex2_3-R	CACCTGAATCCGCGATCTATT	
VPS35-ex2-F	TACAGGCTGTGAAGGTCCAG	RT-PCR primers for VPS35 ex9 skipping and alternative 3'ss usage
VPS35-ex4-R	AGACCTCCAAGTAGTGCAGT	
SLC9A8-ex8-F	TCTCAACGATGCAGTCTCCA	RT-PCR primers for SLC9A8 ex3 skipping and alternative 3'ss usage
SLC9A8-ex10-R	ATGCCAAACTCCAAGGAAGG	
MAPK12-ex7-F	AGATGACTGGGTACGTGGTG	RT-PCR primers for MAPK12 ex7 skipping
MAPK12-ex9-R	CCCCGTCACCTTCATGATCT	
OCRL-ex21-ex22-F	TTCCTGAGACAATCCCTGGC	RT-PCR primers for OCRL ex21-23 ligated mRNA and intron 21 retention
OCRL-int21-F	CTCTGTGTGGCCTTTCTCCT	
OCRL-ex23-ex22-R	AAGCTGGGAGATCACCTGTC	

Northern blot probes and primer extension oligos

hU12-1-31	GTTATTTTCCTTACTCATAAGTTTAAGGCAT	Probe for U12 snRNA (nucleotides 1-31)
U12tag-1	TAAGGCATGTATCACTATCTCAT	Probe for exogenous tagged U12 snRNA
U12-9L	AGATCGCAACTCCCAGGCATCCCGC	Probe for U12 snRNA
U11-6L	TCTCTTGATGTCGATTCCGCAC	Probe for U11 snRNA
U4atac-11L	AAAATTGCACCAAAATAAAGCAAAA	Probe for U4atac snRNA
U6atac-10L	AAAAACGATGGTTAGATGCCACGAA	Probe for U6atac snRNA
U1-1L	GCAGTCCCCCACTACCACAAATTAT	Probe for U1 snRNA
U2-3L	TTTAATATATTGTCCTCGGATAGAG	Probe for U2 snRNA
U4-1L	TCACGGCGGGGTATTGGGAAAAGTT	Probe for U4 snRNA
U6-3L	AATATGGAACGCTTCACGAATTTGC	Probe for U6 snRNA
hU1-50-70	TGCAATGGATAAGCCTCGCCC	Probe for U1 snRNA
hU2-35-66	TATCAGATATTAAACTGATAAGAACAGATACT	Probe for U2 snRNA
hU4-27-47	GCCTCGGATAGACCTCATTGG	Probe for U4 snRNA

hU5-56-85	GGTTAAGACTCAGAGTTGTTCCTCTCCACG	Probe for U6 snRNA
h7SK-1	GTGTCTGGAGTCTTGGAAGC	Probe for 7SK snRNA
tRNA(Glu)	CCGGGAATCGAACCCGGGCCGCGGCGGTGAGA GCGCCGAATCCTAACCAC	Probe for tRNA(Glu)
F30-2xdBroccoli-1	TACGAATATCTGGACCCGACCG	Probe for F30-2xdBroccoli aptamer
U12-RT-LNA-17	CGGGCAGATCGCAACTC	Primer extension oligo for U12 snRNA
**Oligos for cloning U12 snRNA fragments**		
hU12_1-23_B	CCTTACTCATAAGTTTAAGGCAT/iBiodT/T /3Bio/	Biotinylated oligo for biotin-streptavidin pulldown of U12 snRNA
S-RNA_cFP_loop	/5Phos/NNGATCGTCGGACTGTAGAACTCTG AACtttt /ideoxyU/GTTCAGAGTTCTAC	Oligo ligated to 3’ end of RNA (pre-adenylated)
TruSeqP7	ACGTGTGCTCTTCCGATCTNNNNNNNN/3AmMO /	Annealed and ligated to 3’ end of cDNA
TruSeqP7c	/5Phos/AGATCGGAAGAGCACACGTCTGAAC TCCAGTCAC	
U12-lig1	CAGAGTTCTACAGTCCGACGA	Primers used for RT-PCR step
U12-lig2	GTGCTCTTCCGATCTATGCC	
**siRNAs**		
siCTRL	not disclosed by the manufacturer	Negative control siRNA (MISSION siRNA Universal Negative Control #1, Sigma-Aldrich SIC001)
siMTR4	CAAUUAAGGCUCUGAGUAAdTdT (sense) /5Phos/UUACUCAGAGCCUUAAUUGdTdT (antisense)	siRNA for MTR4 (Custom ON-TARGETplus siRNA, Dharmacon)
siRBM7	GCGUAAAGUCAGAAUGAAUdTdT (sense) /5Phos/AUUCAUUCUGACUUUACGCdTdT (antisense)	siRNA for RBM7 (Custom ON-TARGETplus siRNA, Dharmacon)
siTOE1	CUGAAAAUGUCUUGGGAAAdTdT (sense) /5Phos/UUUCCCAAGACAUUUUCAGdTdT (antisense)	siRNA for TOE1 (Custom ON-TARGETplus siRNA, Dharmacon)
Nucleotides in the 5’ overhang of primers are in lowercase. Restriction sites are underlined. LNA nucleotides are marked in bold. The following are modification codes used by IDT: /iBiodT/ = internal biotin-dT, /3Bio/ = 3’ biotin, /5Phos/ = 5’ phosphorylation, /ideoxyU/ = internal deoxyuridine, /3AmMO/ = 3' amino modifier.		

Supplementary Table S2. Variants in the RNU12 gene from the gnomAD database (v3.1)

Table provided as a separate file (SupplementaryTableS2+S3.xlsx).

Supplementary Table S3. Predicted effect of single-nucleotide variants in U12 snRNA on RNA structure.

Table provided as a separate file (SupplementaryTableS2+S3.xlsx).
